# *Limosilactobacillus* (*Lactobacillus*) *fermentum* ALAL020, a Probiotic Candidate Bacterium, Produces a Cyclic Dipeptide That Suppresses the Periodontal Pathogens *Porphyromonas gingivalis* and *Prevotella intermedia*

**DOI:** 10.3389/fcimb.2022.804334

**Published:** 2022-03-07

**Authors:** Tomomi Kawai, Tomoko Ohshima, Takeshi Tanaka, Satoshi Ikawa, Atsushi Tani, Naoya Inazumi, Ryoichi Shin, Yukie Itoh, Karen Meyer, Nobuko Maeda

**Affiliations:** ^1^ Department of Oral Microbiology, School of Dental Medicine, Tsurumi University, Yokohama, Japan; ^2^ Research Division of Polymer Functional Materials, Osaka Research Institute of Industrial Science and Technology, Izumi, Japan; ^3^ Research Division of Applied Material Chemistry, Osaka Research Institute of Industrial Science and Technology, Izumi, Japan; ^4^ Graduate School of Human Development and Environment, Kobe University, Kobe, Japan; ^5^ Technical Support Division, Graduate School of Science, Osaka University, Toyonaka, Japan; ^6^ Research Institute for Fermentative Microbes, A. L. A. Corporation, Tokyo, Japan; ^7^ Department of Dental Hygiene, Tsurumi Junior College, Yokohama, Japan

**Keywords:** antibacterial peptide, periodontal pathogen, cyclic dipeptide, probiotics, nuclear magnetic resonance analysis, chemical synthesis

## Abstract

Periodontal disease develops as a result of oral microbiota in dysbiosis, followed by the growth of periodontal pathogens such as *Porphyromonas gingivalis* and *Prevotella intermedia*. In case of acute symptoms, antibacterial agents and disinfectants are administered, however the appearance of drug-resistant bacteria and allergies cause problems. In recent years, studies on the effects of probiotics have been conducted as an alternative therapy for periodontitis. However, the basic mechanism of the inhibitory effect of probiotic bacteria on periodontal disease has not been clearly elucidated. To clarify the antibacterial mechanism of probiotics against periodontal pathogens, we used *Limosilactobacillus (Lactobacillus) fermentum* ALAL020, which showed the strongest antibacterial activity against *P. gingivalis* and *P. intermedia* among 50 screened lactic acid bacteria strains. The antibacterial substances produced were identified and structurally analyzed. After neutralizing the MRS liquid culture supernatant of ALAL020 strain, the molecular weight (m/z) of the main antibacterial substance separated by gel filtration column chromatography and reverse phase HPLC was 226.131. This low molecular weight compound was analyzed by LC-MS and disclosed the composition formula C_11_H_18_O_3_N_2_, however the molecular structure remained unknown. Then, structural analysis by NMR revealed C_11_H_18_O_3_N_2_ as the cyclic dipeptide, “hexahydro-7-hydroxy-3- (2-methylpropyl) pyrrolo [1,2-a] pyrazine-1,4-dion cyclo (Hyp-Leu) “. Based on the results of this analysis, cyclo (Hyp-Leu) was chemically synthesized and the antibacterial activity against *P. gingivalis* and *P. intermedia* was measured. The minimum inhibitory concentration (MIC) was 2.5 g/L and the minimum bactericidal concentration (MBC) was shown to be less than 5 g/L. In addition, an *in vitro* epithelial tissue irritation test at 10 g/L showed no tissue toxicity. So far there are no reports of this peptide being produced by probiotic bacteria. Furthermore, antibacterial activity of this cyclic dipeptide against periodontal disease bacteria has not been confirmed. The results of this study might lead to a comprehensive understanding of the antibacterial mechanism against periodontal disease bacteria in future, and are considered applicable for the prevention of periodontal disease.

## 1 Introduction

Periodontitis is an infectious disease and one of the two major dental diseases along with caries. It is caused by periodontal bacteria, mostly specific gram-negative anaerobic bacteria, which promote dysbiosis in the oral ecosystem (Houle and Grenier, 2003). *Porphyromonas gingivalis* and *Prevotella intermedia* are two of the three major periodontal pathogens and species, which were reclassified from the genus *Bacteroides* (Taniguchi et al., 1998). *P. gingivalis* is considered to be the most important periodontal pathogen. It is frequently isolated from patients with chronic periodontitis and has the strongest periodontal pathogenicity among oral bacteria (Bostanci and Belibasakis, 2012; Darveau et al., 2012). *P. intermedia* is not only a cause of chronic periodontitis, but also of gestational gingival inflammation, using female hormones as a growth factor (Kornman & Loesche, 1982), and it is also a causative agent of acute necrotizing ulcerative gingivitis (Bolivar et al., 2012).

Antibacterial agents and disinfectants are used for treatment, but also to prevent the onset and recurrence of periodontal disease. However, adverse events, such as the possibility of developing allergies and the emergence of drug-resistant bacteria are problematic (Watanabe, 1966; Pradier et al., 1997; van Winkelhoff et al., 2000). In addition, periodontal pathogens are present in plaque formed by indigenous bacteria in the oral cavity, and removing all indigenous bacteria leads to the onset of bacterial alternation, which in turn harms health. Therefore, the best preventive method is to eliminate only harmful pathogens, as well as dysbiosis, and to improve the microbiota.

In recent years, attention has been focused on the usefulness of probiotics represented by lactic acid bacteria in the treatment and prevention of periodontal disease (Gupta, 2011). According to Fuller probiotics are defined as “living microbes that improve the gut microbiota and have beneficial health effect to the host”. (Fuller, 1989). Attempts have been made to prevent oral diseases such as dental caries and periodontal disease by applying probiotics directly into the oral cavity (Krasse et al., 2006; Riccia et al., 2007; Vivekananda et al., 2010). For example, Ishikawa et al. (2003) reported that 4-week oral administration of the *Ligilactobacillus (Lactobacillus) salivarius* TI2711 strain significantly reduced the major periodontopathic bacteria, *P. gingivalis* and *P. intermedia.* Vivekananda et al. reported that the viable strains of *Limosilactobacillus (Lactobacillus) reuteri* DSM17938 and ATCC PTA5289 reduce the number of oral *P. gingivalis* in patients with periodontal disease. However, there are only few reports on the mechanism and substances of probiotics against oral bacteria.

Organic acids such as lactic acid and acetic acid, hydrogen peroxide, and bacteriocin have been reported as antibacterial substances produced by lactic acid bacteria. Takahashi et al. reported that *P. gingivalis* is highly acid-sensitive and growth is suppressed below pH 6.5 (Takahashi et al., 1997). Matsuoka et al. reported that the antibacterial activity of the *Ligilactobacillus salivarius* TI2711 strain against *P. gingivalis* is due to lactic acid (Matsuoka et al., 2004). However, maintaining a low pH state in the oral cavity with acid is not favored because it may induce caries and hyperesthesia. In order to solve this problem, it is preferred to apply a different antibacterial substance produced by probiotic bacteria for the treatment and prevention of periodontal disease.

In a previously reported study (Kawai et al., 2016), we selected *L. fermentum* ALAL020 from 50 *Lactobacillus* strains, which showed strong antibacterial properties against *P. gingivalis* in a neutral environment, and the active ingredient in the culture supernatant was extracted. When the active ingredient was fractionated and purified by high performance liquid chromatography (HPLC) using a reverse phase column, a low molecular weight substance consisting of two very adjacent peaks was obtained, which was analyzed using LC-MS. However, both peaks were found to be antibacterial substances with a molecular weight of 226.131 and a molecular formula of C_11_H_18_O_3_N_2_ (Kawai et al., 2016). Since the kind of molecular structure and properties were unclear in this study, we conducted a detailed structural analysis of the antibacterial substance C_11_H_18_O_3_N_2_, and confirmed the biological property of antibacterial activity and safety for human tissues.

## 2 Materials and Methods

### 2.1 Preparation of Analytical Samples

We used *L. fermentum* ALAL020, a strain derived from fermented soymilk and provided by the Research Institute for Fermentative Microbes, A. L. A. Corporation. After suspending *L. fermentum* ALAL020 in Man-Rogosa-Sharpe (MRS) broth (Difco, Becton Dickinson and Company, Sparks, MD, USA), the bacteria were cultured in an anaerobic incubator (BACTRON, Sheldon Manufacturing Inc., Portland, OR, USA) in an environment of 80% N_2_, 10% CO_2_ and 10% H_2_ at 37°C for 24 hours.

The culture solution was centrifuged at 7000 x g for 20 minutes to prepare a culture supernatant sample. Four times volume of acetone was added to the culture supernatant, and the mixture was separated into supernatant and precipitate. The acetone extract supernatant was used as a water-soluble fraction and lyophilized. The total volume was dissolved in 150 ml of water and 1/7 volume of 21 ml was placed on a Sephadex G-25 column and eluted with ion-exchange water. The obtained fraction with antibacterial property was lyophilized and dissolved to a concentration of 100 g/L. 1/63 amount was applied to HPLC in 10 batches and analyzed by high performance liquid chromatography (HPLC), using a Wakosil II 5C18 AR Prep (20.0 mmφ x 250 mm) column, with a flow velocity of 5.0 ml/min and a concentration gradient of 10-50% acetonitrile containing 0.05% trifluoroacetic acid. The gradient was applied for 30 minutes and the elute was obtained in 11 fractions (H1-H11). Two fractions, H8-1 and H8-2 with adjacent peaks and antibacterial properties were confirmed at an absorbance of 210 nm (Kawai et al., 2016). H8 fraction was lyophilized and then dissolved in 0.6 mL of de-ionized pure water. After adjusting to 20 g/L, it was used for MS and NMR analysis.

### 2.2 Nuclear Magnetic Resonance (NMR) Analysis

For the structural analysis of the purified product in H8 fraction, an NMR device (AVANCE700, Bruker Biospin) was used with resonance frequency of 700.333 MHz at a temperature of 10°C. ^1^H NMR and ^13^C NMR were measured in the first dimension, and ^1^H-^1^H COSY (correlation spectroscopy), ^1^H-^13^C HSQC (hetero nuclear single-quantum correlation), and ^1^H-^13^C HMBC (hetero nuclear multiple bond correlation) were measured in the second dimension. Heavy water or deuterated acetone were used as solvent. A JNM-500A NMR spectrometer (Japan Electron Optics Laboratory) was used as the measuring instrument for the NMR analysis of chemically synthesized peptides. Heavy water or deuterated chloroform was used as the solvent, and the measurement was performed with resonance frequency of 500.00 MHz at a temperature of 20 °C.

### 2.3 Synthesis of the Cyclic DiPeptide (L-Hyp-L-Leu)

All chemicals, reagents and solvents were used without further purification.

Condensation with COMU (El-Faham et al., 2009; El-Faham and Albericio, 2010), removal of the Boc (*t*-butoxycarbonyl) group (Lundt et al., 1978), intramolecular cyclization (Sollis, 2005) and removal of the Bn (benzyl) group (Hawker et al., 1992) were carried out in sequence. The details are as follows. Dimethylaminomorpholino uronium hexafluorophosphate (COMU) (1.028 g, 2.4 mmol) was added to a dimethylformamide (super dehydrated) solution (10 mL) in a round bottom flask at -10°C containing Boc-*O*-benzyl-L-hydroxyproline **2** (643 mg, 2.0 mmol), L-Leucine methyl ester hydrochloride **3** (727 mg, 4.0 mmol) and diisopropylethylamine (1.7 mL, 9.8 mmol) 1-[(1-(Cyano-2-ethoxy-2-oxoethylideneaminooxy). The mixture was stirred under nitrogen at the same temperature for 30 minutes. Then the mixture was continuously stirred at room temperature for 18 hours and diluted with a hexane/ethyl acetate (7/3) solution. The resulting mixture was washed with a saturated aq. NaHCO_3_, and the layers were separated. The organic layer was dried over Na_2_SO_4_ and concentrated *via* rotary evaporation. The residue was passed through a short column (silica gel, hexane/ethyl acetate = 7/3) to obtain the crude condensation product **4** (1.031 g) as a yellow oil.

To a dichloromethane solution (10 mL) in a round bottom flask containing the crude condensation product **4** (1.031 g) trifluoroacetic acid (1.5 mL, 20 mmol) was slowly added at room temperature under air and the mixture was stirred for 4 hours. Thereafter, a saturated aq. NaHCO_3_ was poured into the mixture. The layers were separated and extracted with chloroform. The organic extract was dried over Na_2_SO_4_ and concentrated in vacuo to obtain the deprotected product **5** (604 mg, 87% in 2 steps) as a yellow oil.

To a dimethylformamid solution (7 mL) in a round bottom flask containing the deprotected product **5** (604 mg, 1.8 mmol) piperidine (1.7 mL, 17 mmol) was added at room temperature under air and the mixture was stirred at 70°C for 12 hours. Thereafter, it was left at room temperature. Then, the mixture was concentrated in vacuo to obtain the cyclic product **6** (511 mg, 92%) as a yellow solid.

To a tetrahydrofuran (super dehydrated, stabilizer free) solution (16 mL) in a round bottom flask containing the cyclic product **6** (511 mg, 1.6 mmol) 10 wt% palladium on carbon (511 mg, 100 wt%) was added at room temperature under air and the flask was charged with hydrogen using a hydrogen filled balloon. The mixture was stirred at 55 °C for 5 hours. The balloon was removed, and the flask was left at room temperature. After dilution with methanol, a suction filtration through filter paper was carried out to eliminate the palladium carbon. The collected solution was concentrated in vacuo, and then the obtained powder was rinsed with diethyl ether to get cyclo (L-Hyp-L-Leu) **1** (283 mg, 78%).

### 2.4 LC-MS Analysis

An HPLC-purified sample was analyzed using an Ultimate 3000 UHPLC System (Thermo Fisher Scientific, Inc., Waltham, MA, USA), and a Shim-pack VP-ODS column (150 mm × φ4.6 mm, Shimadzu, Tokyo, Japan). Gradient elution was performed using 10-50% acetonitrile with 0.05% formic acid. An MS analysis was performed with a Mass Spectrometry Q Exactive ^TM^ Quadrupole/Orbitrap hybrid mass spectrometer (Thermo Fisher Scientific). Ionization was performed using the ESI method and detection in the positive mode.

### 2.5 Antimicrobial Test

The antibacterial test with the peak fractions was performed according to the method described in a previous report (Kawai et al., 2016) using *P. gingivalis* ATCC 33277 as the index bacterium.

The antibacterial activity of the synthetic dipeptide cyclo (Hyp-Leu) against the *P. gingivalis* reference strain (ATCC 33277) and the *P. intermedia* reference strain (ATCC 25611) was evaluated as the minimum inhibitory concentration (MIC) and the minimum bactericidal concentration (MBC, measuring colony forming units CFU).

-80°C cryopreserved cells of *P. gingivalis* and *P. intermedia* were thawed and suspended in brain heart infusion (BHI) medium (Difco, Becton Dickinson, Maryland, USA), supplemented with 5 μg/ml hemin, 1 μg/mL menadione, 0.5% yeast extract and 0.05% cysteine hydrochloride (Wako Pure Chemical Industries, Ltd., Osaka), and cultured in an anaerobic incubator (BACTRON, Sheldon Manufacturing Inc.) under the atmosphere of 80% N_2_, 10% CO_2_, 10% H_2_ at 37°C. After culturing for 2 days, the bacteria were sub-cultured in fresh medium for another 2 days to gain 10^7^ CFU/ml each, which were used as the test bacteria.

40 μL of BHI medium supplemented with 5 μg/mL hemin and 1 μg/mL menadione were dispensed into a 96-well plate, and 5 μL of each 10 g/L, 5 g/L, and 2.5 g/L aqueous solution of the synthetic peptide was added. 20 g/L, 10 g/L, 5 g/L and 2.5 g/L sodium lactate (Lactate Na) were used as control series. 5 μL of the two test bacterium culture solutions were inoculated and cultured for 2 days under anaerobic conditions at 37 °C. The turbidity was measured at 650 nm, and a value of 0.15 or less corresponds to MIC. After MIC determination, the culture medium was inoculated on Brucella HK agar medium (KYOKUTO PHARMACEUTICAL INDUSTRIAL CO., Tokyo, Japan) supplemented with 5% sheep de-fibered blood by plating, and then anaerobically cultured at 37 °C for 5 days. MBC was determined by measuring CFU.

### 2.6 Epithelial Tissue Irritation Test

Epithelial stimulation tests were performed according to the standard protocol (OECD TG 439) using *in vitro* reconstructed human epidermal models (EPI-200SIT, KURABO, Osaka, Japan). A 10 g/L cyclo (Hyp-Leu) dipeptide aqueous solution was used as the test solution, PBS (phosphate buffered saline) as a negative control and 5% SDS (sodium dodecyl sulfate) as a positive control. The metabolic activity of the treated tissue was measured by the manufacturer specified MTT assay and compared to the described criteria.

The obtained data were statistically analyzed using IBN SPSS Statistics version 19 (IBM, Armonk, NY, USA). Comparisons between the three groups were analyzed by the Kruskal-Wallis test, and data on synthetic dipeptides compared to the positive controls were analyzed by the Mann-Whitney U test.

## 3 Results

### 3.1 Physicochemical Property Analysis of the Compound and Synthesis

#### 3.1.1 NMR Analysis

**Figure 1:**
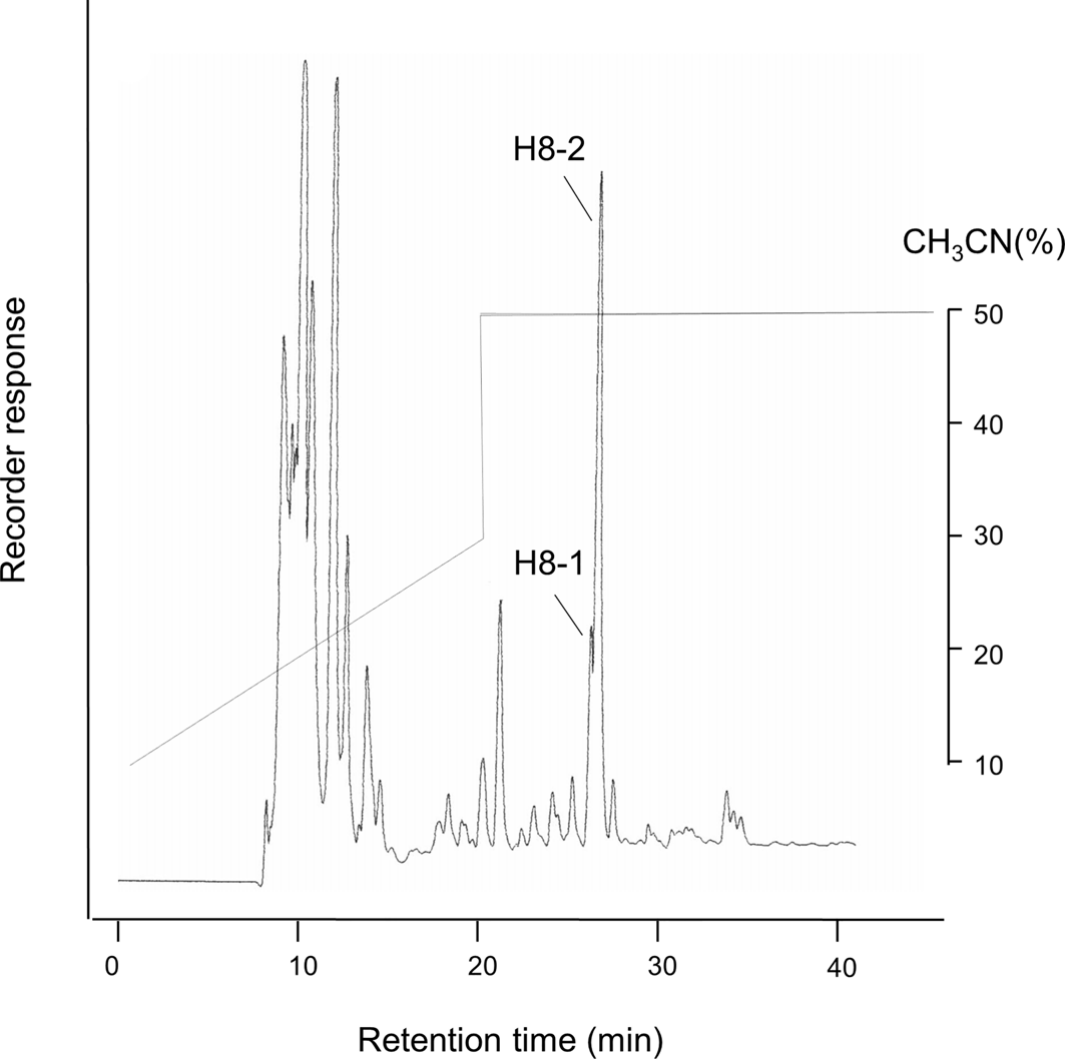
HPLC fractions of *L. fermentum* ALAL020 culture supernatant. The culture supernatant was fractionated with reverse-phase chromatography by HPLC on an ODS (C18) column.

As in the previous report (Kawai et al., 2016), each peak was divided into 11 fractions, with H8-1 and H8-2 very adjacent (**Figure 1**). According to the results of the antibacterial tests against *P. gingivalis*, fraction H8-1 had the highest antibacterial activity, followed by H8-2. The active fractions (H8-1 and H8-2) corresponded to 9.7% of the eluted fraction and a concentration of 0.47 g/L.

It has also been confirmed that the molecular weights and formulas of the fractions H8-1 and H8-2 were both 226.131 Da and C_11_H_18_N_2_O_3_ respectively (Kawai et al., 2016).

**Figure 2:**
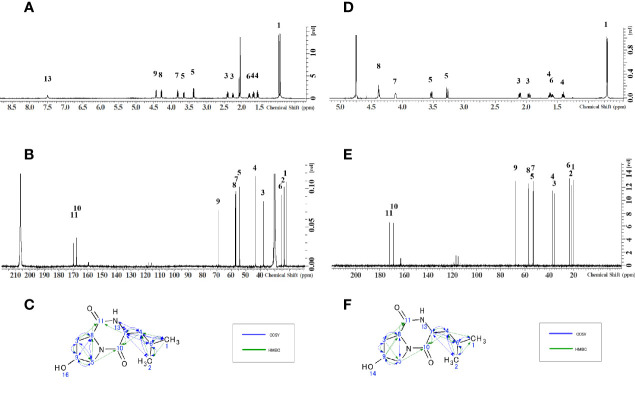
Structure determination by NMR. **(A)** ^1^H-NMR of H8-1, **(B)** ^13^C-NMR of H8-1, **(C)** COSY and HMBC correlations of H8-1, **(D)** ^1^H-NMR of H8-2, **(E)** ^13^C-NMR of H8-2, **(F)** COSY and HMBC correlations of H8-2.

The NMR spectra obtained for the antibacterial substance of H8-1 are shown in **Table 1**. The ^1^H NMR spectrum showed one NH resonance (δH: 7.49 (^1^H, s)), two methyl signals (δH: 0.92 (^3^H, d, J = 6.6Hz), 0.96 (^3^H, d, J = 6.7)), six methylene resonances (δH: 2.22-2.27 (^1^H, m), 2.38-2.42 (^1^H, m), 1.65-1.69 (^1^H, m), 1.53-1.57 (^1^H, m), 3.37 (^1^H, dd, J = 11.9, 5.4Hz), 3.65 (^1^H, ddd, J = 11.9, 3.7, 0.8Hz)) and four methine signals (δH: 1.75-1.82 (^1^H, m), 3.81-3.84 (^1^H, m), 4.28 (^1^H, dd, J = 8.6, 7.1Hz), 4.42-4.45 (^1^H, m)) (**Figure 2A**). The ^13^C NMR spectrum showed 11 C atoms and carbon signals (δC: 21.83, 23.17, 25.01, 37.61, 43.09, 53.90, 56.46, 56.91, 68.46, 167.28, 169.29) (**Figure 2B**). Comprehensive analysis results of ^1^H-^1^H COSY, ^1^H-^13^C HSQC and ^1^H-^13^C HMBC by two-dimensional NMR analysis (SF-A, B, C) revealed two amino acids, hydroxyproline and leucine (**Figure 2C**).

**Table 1:** Chemical shifts and 2D correlations of NMR spectra in H8-1.

#	Atom#	C Shift	XHn	H Shift	H Multiplicity	COSY	H HMBC	C HMBC
**1**	**1**	**21.83**	**CH3**	**0.92**	**d**	**6**	**2, 4, 4, 6**	**2, 6, 4**
**2**	**2**	**23.17**	**CH3**	**0.96**	**d**	**6**	**1, 4, 4**	**1, 6, 4**
**3**	**6**	**25.01**	**CH**	**1.79**	**m**	**1, 2, 4**	**1, 2, 4, 4**	**1**
**4**	**3**	**37.61**	**CH2**	**2.22-2.27**	**m**	**3, 8, 9**	**5, 8**	**8, 9, 11**
**5**	**3**	**37.61**	**CH2**	**2.38-2.42**	**m**	**3, 8, 9**	**5, 8**	**5, 8, 9, 11**
**6**	**4**	**43.09**	**CH2**	**1.65-1.69**	**m**	**4, 6, 7**	**1, 2, 7**	**1, 2, 6, 7, 10**
**7**	**4**	**43.09**	**CH2**	**1.53-1.57**	**m**	**4, 6, 7**	**1, 2, 7**	**1, 2, 6, 7, 10**
**8**	**5**	**53.90**	**CH2**	**3.37**	**dd**	**5, 9**	**3**	**3, 10**
**9**	**5**	**53.90**	**CH2**	**3.65**	**ddd**	**5, 9**	**3**	**8, 9**
**10**	**7**	**56.46**	**CH**	**3.81-3.84**	**m**	**4, 4, 13**	**4, 4**	**4, 10, 11**
**11**	**8**	**56.91**	**CH**	**4.28**	**dd**	**3, 3**	**3, 3, 5**	**3, 11**
**12**	**9**	**68.46**	**CH**	**4.42-4.45**	**m**	**3, 3, 5, 5**	**3, 3, 5**	
**13**	**10**	**167.28**	**C**				**4, 4, 5, 7**	
**14**	**11**	**169.29**	**C**				**3, 3, 7, 8**	
**15**	**13**		**NH**	**7.49**	**s**	**7**		

The NMR spectra obtained for the antibacterial substance H8-2 are shown in **Table 2**. The ^1^H NMR spectrum showed one NH resonance (δH ): two methyl signals (δH: 0.75 (^6^H, d, J = 6.4Hz)), six methylene resonances (δH: 2.15 (^1^H, dd, J = 13.5, 6.4 Hz)), 1.98-2.02 (^1^H, m), 1.64-1.69 (^1^H, m), 1.43-1.47 (^1^H, m), 3.57 (^1^H, dd, J = 13.2, 4.4Hz), 3.31 (^1^H, d, J = 13.2Hz)) and four methine signals (δH: 1.60-1.65 (^1^H, m), 4.15-4.16 (^1^H, m), 4.41-4.42 (^1^H, m), 4.43 (^1^H, t, J = 4.5Hz)) (**Figure 2D**).

**Table 2:** Chemical shifts and 2D correlations of NMR spectra in H8-2.

#	Atom#	C Shift	XHn	H Shift	H Multiplicity	COSY	H HMBC	C HMBC
**1**	**1**	**20.61**	**CH3**	**0.75**	**d**	**6**	**2, 4, 4, 6**	**2, 6, 4**
**2**	**2**	**21.99**	**CH3**	**0.75**	**d**	**6**	**1, 4, 4, 6**	**1, 6, 4**
**3**	**6**	**23.72**	**CH**	**1.60-1.65**	**m**	**1, 2**	**1, 2, 4, 4, 7**	**1, 2, 4, 7**
**4**	**3**	**35.92**	**CH2**	**2.15**	**dd**	**3, 8, 9**	**5, 5, 8**	**8, 9, 11**
**5**	**3**	**35.92**	**CH2**	**1.98-2.02**	**m**	**3, 8, 9**	**5, 5, 8**	**5, 9**
**6**	**4**	**37.22**	**CH2**	**1.64-1.69**	**m**	**4, 7**	**1, 2, 6, 7**	**1, 2, 6, 7, 10**
**7**	**4**	**37.22**	**CH2**	**1.43-1.47**	**m**	**4, 7**	**1, 2, 6, 7**	**1, 2, 6, 7, 10**
**8**	**7**	**53.23**	**CH**	**4.15-4.16**	**m**	**4, 4**	**4, 4, 6**	**6, 4, 10**
**9**	**5**	**53.61**	**CH2**	**3.57**	**dd**	**5, 9**	**3, 8, 9**	**3, 8, 9, 10**
**10**	**5**	**53.61**	**CH2**	**3.31**	**d**	**5**	**3, 8, 9**	**3, 8, 10**
**11**	**8**	**57.04**	**CH**	**4.41-4.42**	**m**	**3, 3**	**3, 5, 5, 9**	**3, 5, 11**
**12**	**9**	**67.68**	**CH**	**4.43**	**t**	**3, 3, 5**	**3, 3, 5**	**3, 5, 8, 11**
**13**	**10**	**168.58**	**C**				**4, 4, 5, 5, 7**	
**14**	**11**	**172.23**	**C**				**3, 8, 9**	

The ^13^C NMR spectrum showed 11 C atoms and carbon signals (δC: 20.61, 21.99, 23.72, 35.92, 37.22, 53.23, 53.61, 57.04, 67.68, 168.58, 172.23) (**Figure 2E**). The result of comprehensive two-dimensional NMR analysis ^1^H-^1^H COSY, ^1^H-^13^C HSQC and ^1^H-^13^C HMBC showed two amino acids, hydroxyproline and leucine, similar to H8-1 (**Figure 2F**).

**Figure 3:**
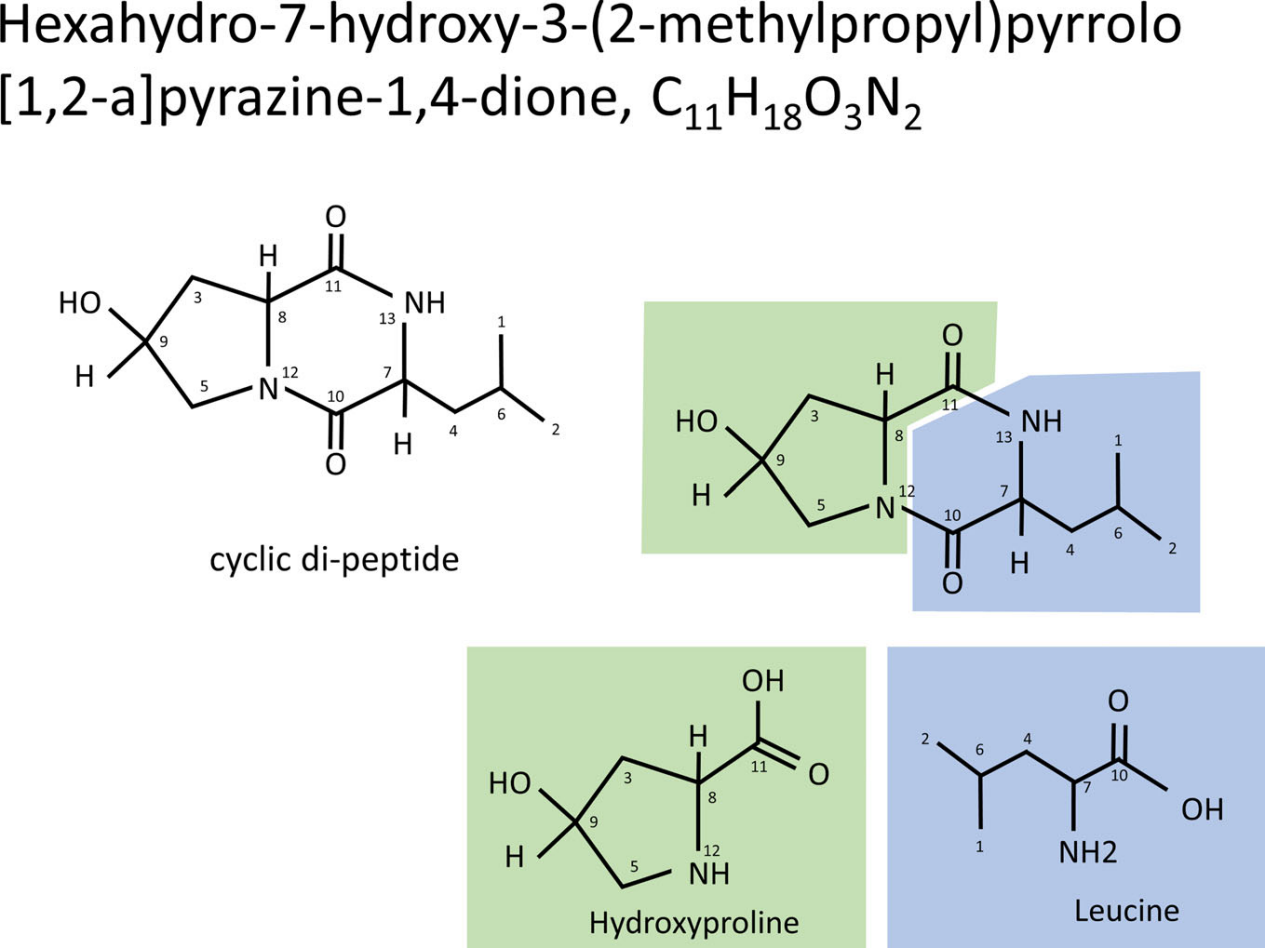
Structure of cyclo (Hyp-Leu). From the result of NMR analysis, the molecule of C_11_H_18_O_3_N_2_ was revealed to form a cyclic di-peptide of hydroxyproline and leucine.

As a result of the above, the antibacterial substances corresponding to fractions H8-1 and H8-2 are both hexahydro-7-hydroxy-3- (2-metylpropyl) pyrrolo [1,2- a] pyrazine-1,4-dion as illustrated in the structural formula of **Figure 3**. That means, it is a cyclic dipeptide of hydroxyproline and leucine, abbreviated as “cyclo (Hyp-Leu)” (**Figure 3**).

#### 3.1.2 Synthesis and Structural Analysis of Cyclo (L-Hyp-L-Leu)

**Figure 4:**
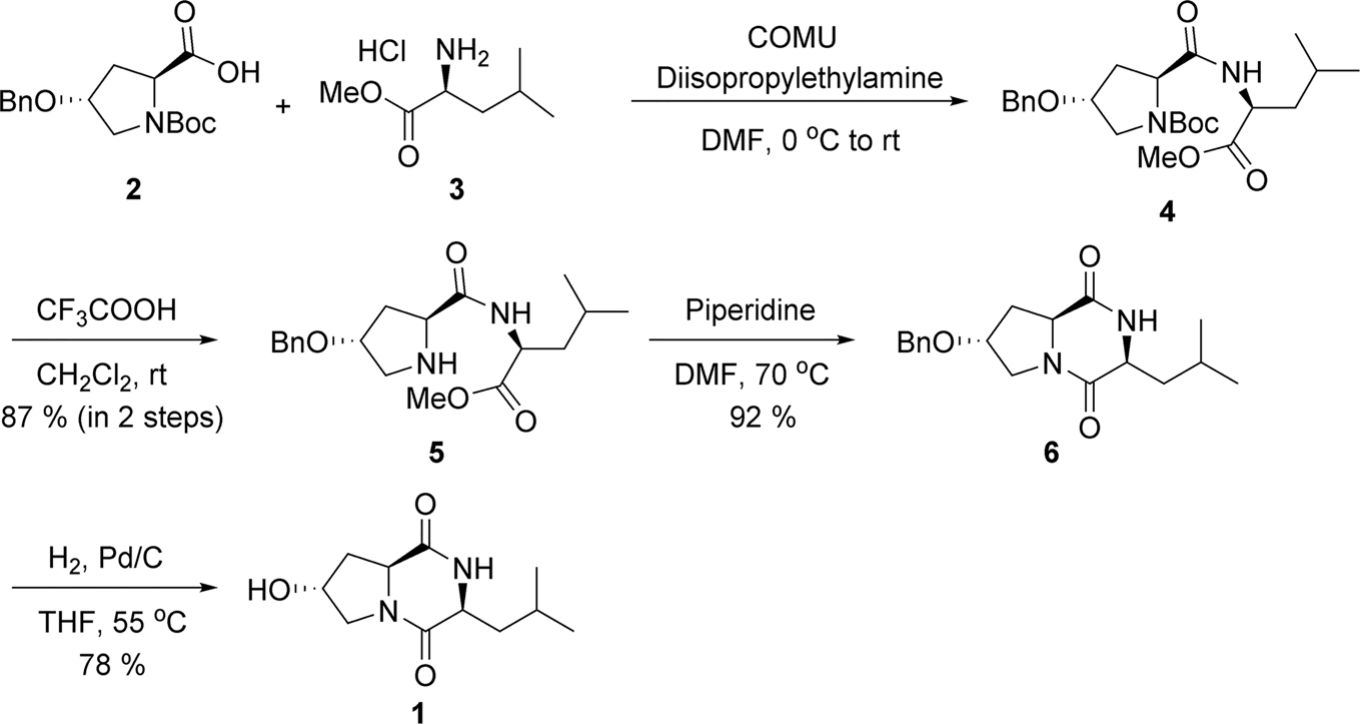
Scheme: Synthetic pathway of cyclo L-Hyp-L-Leu.

Composition and structure of the cyclic dipeptide Hyp-Leu (hexahydro-7-hydroxy-3- (2-metylpropyl) pyrrolo [1,2-a] pyrazine-1,4-dion) were clarified followed by chemical synthesis. According to the scheme in **Figure 4**, the condensation of commercially available Boc-*O*-benzyl-L-hydroxyproline (**2** in **Figure 4**) and L-leucine methyl ester hydrochloride (**3** in **Figure 4**) was carried out with COMU as a condensing agent. The Boc group of the condensation product (**4** in **Figure 4**) was then removed by treatment with trifluoroacetic acid, and **5** of **Figure 4** was obtained. The structure was confirmed by NMR analysis (SF-D). ^1^H NMR (CDCl_3_) δ7.94 (^1^H, d, J = 8.5 Hz), 7.34-7.25 (^5^H, m), 4.57-4.52 (^1^H, m), 4.51-4.41 (^2^H, m), 4.08 (^1^H, s), 4.00 (^1^H, t, J = 7.7 Hz), 3.70 (^3^H, s), 3.19 (^1^H, d, J = 12.5 Hz), 2.74 (^1^H, dd, J = 12.5, 3.6 Hz), 2.48-2.43 (^1^H, m), 1.92-1.86 (^1^H, m), 1.67-1.43 (^4^H, m), 0.90 (^6^H, t, J = 5.8 Hz).

The intramolecular cyclization of the amino group and ester group in **5** was carried out under basic condition to form 2, 5-diketopiperazine. The structure was confirmed by NMR analysis (SF-E). ^1^H NMR (CDCl_3_) δ 7.45-7.26 (5H, m), 5.66 (1H, s), 4.52 (2H, s), 4.43 (1H, dd, *J* = 10.8, 6.3 Hz), 4.22-4.20 (1H, m), 4.03-4.01 (1H, m), 3.72 (1H, d, *J* = 13.1 Hz), 3.66 (1H, dd, *J* = 13.1, 4.6 Hz), 2.57 (1H, dd, *J* = 13.5, 6.3 Hz), 2.11-2.01 (2H, m), 1.75-1.46 (2H, m), 0.98 (3H, d, *J* = 6.4 Hz), 0.93 (3H, d, *J* = 6.7 Hz).

Finally, the benzyl group included in **6** was removed by hydrogenolysis to obtain the targeted compound, Cyclo (L-Hyp-L-Leu) (**1** in **Figure 4**). The analyzed structure by NMR was as follows: ^1^H NMR (D_2_O) δ 4.42-4.39 (2H, m), 4.13 (1H, s), 3.56 (1H, dd, *J* = 13.1, 4.6 Hz), 3.30 (1H, d, *J* = 13.1 Hz), 2.14 (1H, dd, *J* = 13.5, 6.4 Hz), 2.01-1.95 (1H, m), 1.70-1.55 (2H, m), 1.46-1.40 (1H, m), 0.73 (6H, d, *J* = 6.4 Hz).

^13^C NMR (D_2_O) δ173.00, 169.33, 68.47, 57.83, 54.41, 54.05, 38.08, 36.74, 24.54, 22.78, 21.46.

**Figure 5:**
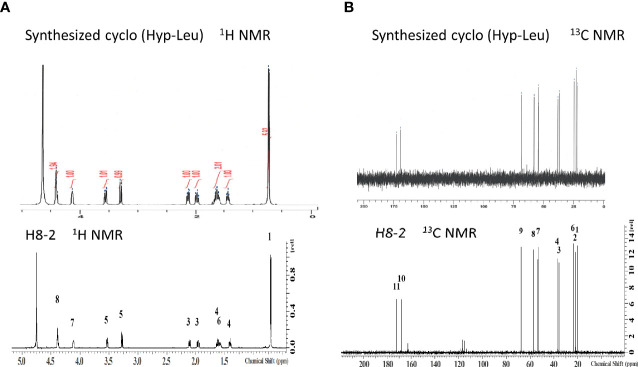
Structure confirmation by NMR. **(A)** ^1^H-NMR spectrum of synthesized cyclo (Hyp-Leu) was matched to that of H8-1, **(B)** ^13^C-NMR spectrum of synthesized cyclo (Hyp-Leu) was matched to H8-1 ^13^C NMR.

When these NMR spectra were compared with the spectra of H8-1 and H8-2 derived from natural products, both ^1^H NMR and ^13^C NMR of H8-2 were in agreement (**Figure 5**). From this result, H8-2 is considered to be cyclo (L-Hyp-L-Leu) or cyclo (D-Hyp-D-Leu).

MS analysis for ${\text{C}}_{11}{\text{H}}_{19}{\text{N}}_{2}{\text{O}}_{3}^{+}{\left[\text{M}+\text{H}\right]}^{+}$ was performed and the monoisotopic mass of the protonated molecule was estimated 227.1405. Furthermore, from the results of mass spectrometry, it was found that the synthetic product was also C_11_H_18_N_2_O_3_, 226.131.

### 3.2 Biological Effect of the Compound

#### 3.2.1 Antibacterial Test of Synthetic Cyclo (Hyp-Leu) Against Periodontal Disease Bacteria

**Figure 6:**
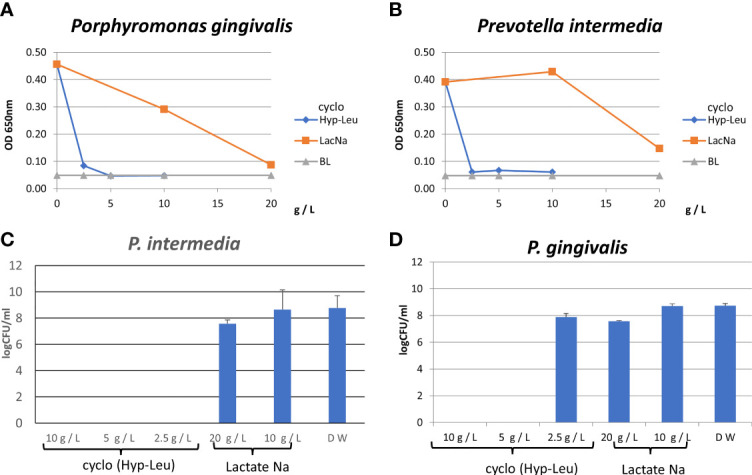
Antibacterial activity of synthesized cyclo (Hyp-Leu). **(A, B)** Show the results of the turbidity assay. BL is medium only. **(C, D)** Show the result of the CFU assay.

Antibacterial tests were conducted against *P. gingivalis* and *P. intermedia*. Synthetic cyclo (Hyp-Leu) showed concentration-dependent antibacterial activity against both bacteria, with a MIC of 2.5 g/L or less (**Figures 5A, B**). Sodium lactate used as a control, is a product of the fermentation process of lactic acid bacteria and a component widely known for its antibacterial properties, however the MIC was 20 g/L (**Figures 6A, B**). Furthermore, examining the MBC of synthetic cyclo (Hyp-Leu), resulted in 5 g/L or less for *P. gingivalis* and 2.5 g/L or less for *P. intermedia* (**Figures 6C, D**).

#### 3.2.2 *In Vitro* Tissue Safety of Synthetic Cyclo (Hyp-Leu)

**Figure 7:**
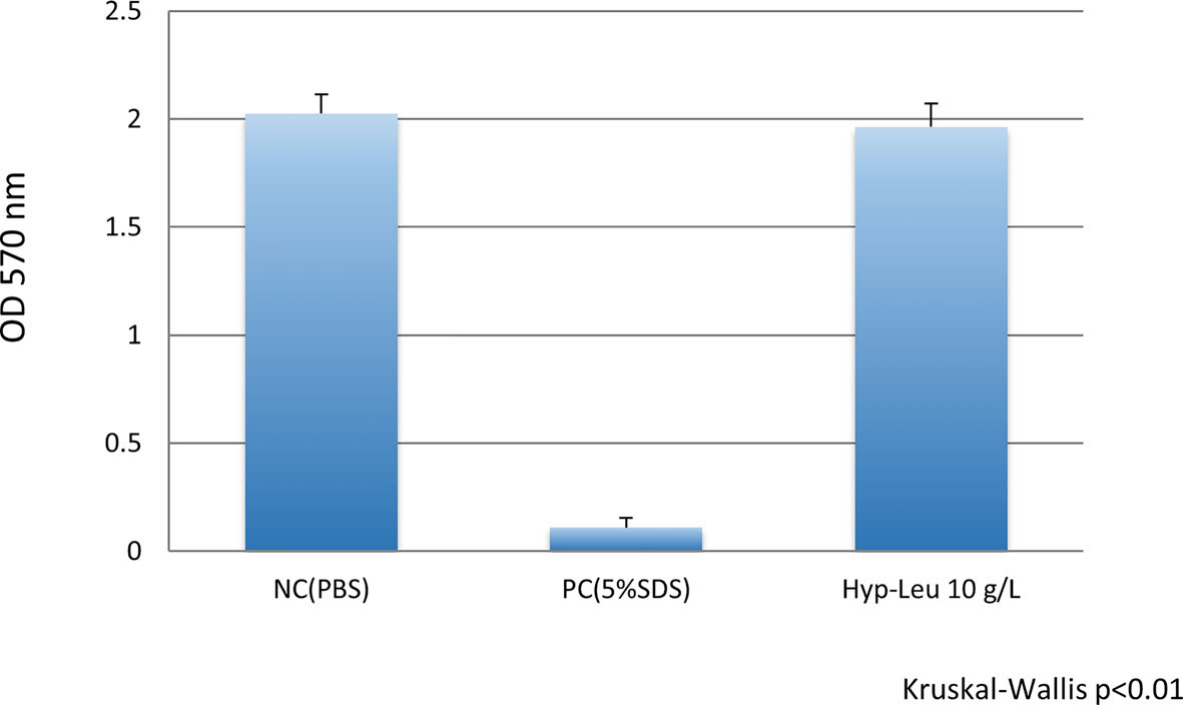
Epithelial tissue toxicity of cyclo (Hyp-Leu). The vertical axis shows the tissue activity level deduced form the result of the MTT assay. NC (negative control, PBS) showed no irritation, and PC (positive control, 5%SDS) showed toxicity. The level of the experimental cyclo (Hyp-Leu) group was almost the same as the NC group and significantly different from the PC group.

The results of the epithelial tissue irritation test are shown in **Figure 7**. The result of the MTT assay was 2.024 ± 0.091 for PBS used as a negative toxicity control. On the other hand, the value of 5.0% SDS used as a positive control indicated a cytotoxic level. The value of the 10 g/L synthetic cyclo (Hyp-Leu) solution was 1.964 ± 0.108, which was an equivalent level to PBS and significantly different from the 5.0% SDS of the positive control (Mann-Whitney U test p> 0.01). Therefore, no toxicity to the epithelial tissue model was observed.

## 4 Discussion

Generally, organic acids such as lactic acid and acetic acid, hydrogen peroxide, and bacteriocin are widely known as bactericidal or antibacterial substances produced by lactic acid bacteria. In addition, several cyclic dipeptides (also known as 2,5-diketopiperazine) have been reported (Klaenhammer, 1988; Piard and Desmazeaud, 1991; Taniguchi et al., 1998; Graz et al., 1999). Cyclic dipeptides are low molecular weight substances obtained by condensing two amino acids. They are expected to be antifungal and antibacterial (Graz et al., 1999). Cyclic dipeptides have been identified in cultures fermented with lactic acid bacteria used in food fermentation (Niku-Paavola et al., 1999; Strom et al., 2002; Magnusson et al., 2003; Ryan et al., 2011). Further, proline-based and leucine-based cyclic dipeptides have been isolated from cultures of multiple lactobacilli. For example, cyclo (L-Pro-L-Pro), cyclo (L-Leu-L-Pro), cyclo (L-Tyr-L-pro), cyclo (L-Met-L-Pro), cyclo (L-His-L-Pro) were identified from *L. amylovorus*, (Ryan et al., 2011), and cyclo (Gly-L-Leu), cyclo (L-Phe-L-Pro) and cyclo (L-Phe-trans-4-OH-L-Pro) have been identified from *L. plantarum* (Niku-Paavola et al., 1999; Strom et al., 2002). Above reports have confirmed that these cyclic dipeptides in lactobacilli cultures have antifungal activity.

The substance hexahydro-7-hydroxy-3- (2-metylpropyl) pyrrolo [1,2-a] pyrazine-1,4-dion was reported by Zhang and Lei (2015), and is a cyclic dipeptide produced by microorganisms of the genus *Streptomyces* classified as soil-derived actinomycetes. It was the same substance as identified in this study. However, as far as we know, the cyclic dipeptides identified in this study have not been reported as substances produced by lactic acid bacteria. This hydrophilic cyclic dipeptide was included in the acetone fraction of the culture supernatant of the *L. fermentum* ALAL020 strain. In a preliminary study, the acetone fraction did not show antibacterial properties against the caries-causing bacterium *Streptococcus mutans* or the oral fungus *Candida albicans* (data not shown), whereas it had excellent antibacterial properties against *P. gingivalis* and *P. intermedia* (**Figure 6**). Since the MIC of cyclo (Hyp-Leu) against *P. gingivalis* and *P. intermedia* was 2.5 mg/mL, the content in the supernatant was about 20% of the MIC. 2014 literature indicates the concentration of a *L. brevis*-produced cyclic dipeptide (Axel et al., 2014) as 10-50 ug/mL, in contrary to *L. fermentum* ALAL020 with 470 μg/mL, which is a production amount 5-10 times higher.

*L. fermentum ALAL020* is derived from fermented food and expected to be effective when used as regular food. This strain is contained in fermented soymilk and shows an immune-stimulatory effect in an *in vitro* assay (unpublished data) as well as an effect of improving liver and kidney disorders in animal model experiments (Shin et al., 2009). Therefore, it can be called a probiotic candidate strain. Clinical efficacy data in humans has to be accumulated in future. In this case, if it exhibits strong bactericidal activity such as antibiotic reagents, it is possible that the bacterial microbiota might be disturbed or a bacterial replacement phenomenon might occur. Therefore, it seems appropriate to compare the antibacterial di-peptide with known antibacterial food substances. For example, the MIC of EGCG (epigallocatechin gallate), a green tea main component, against *P. gingivalis* has been reported to be 0.125-1.0 g/L (0.27-2.2 mM) (Sakanaka et al., 1996; Fournier-Larente et al., 2016). Since MIC of cyclo (Hyp-Leu) was 2.5 g/L (11.1 mM), it is considered the same grade as the green tea component, although the activity is slightly weaker. However, examination with multiple *P. gingivalis* clinical isolates, as shown in the **Supplementary File (E)**, indicate large individual differences in susceptibility, so comparison of the MIC value of single strains is not accurate. Since we have succeeded in peptide synthesis, it seems possible to adjust the activity in future.

Probiotic *L. reuteri* produce reutericyclin with antibacterial property, however this substance tends to be resistant to half of the *E. coli* tested by Gänzle et al. (2004), confirming antibacterial activity only against Gram-positive bacteria. Therefore, reutericyclin is expected to have low activity against periodontopathic bacteria.

MIC of reuterin was reported as 7.5 to 15 mM (5.5-11.1 g/L) in *Escherichia coli* and that of *Bacteroides* 1.9 to 7.5 mM (Cleusix et al., 2007), which means that the anti-bacterial effect is almost at the same level as cyclo (Hyp-Leu) (MIC: 11.1mM) against *P. gingivalis* and *P. prevotella*. In their report, periodontal disease bacteria have not been investigated, but they might have a tendency similar to the closely related genus *Bacteroides*. It was reported that chemically synthesis of reuterin is very difficult because reuterin is an intermediate metabolite and degrades rapidly to various other components such as 3-Hydroxypropionaldehyde (3-HPA) hydrate, HPA dimer, and acrolein (Kang, 2014; Fujiwara et al., 2017). With this unstable property, the clinical application of reuterin seems difficult. Fujiwara et al. synthesized structural derivatives and obtained highly active and stable ones against periodontal bacteria (Fujiwara et al., 2017). The significance of our structural analysis of cyclo (Hyp-Leu) is not only to clarify the mechanism of action in the future, but also to obtain derivatives showing even better activity.

Although the effects of *P. gingivalis* on cell adhesion and invasion have not been investigated, it seems that bactericidal action is effective before adhesion and invasion. Further, it has been confirmed that the trypsin-like enzyme (Gingipain) activity produced by *P. gingivalis*, is not inhibited (data not shown), however it can be said that this is not a necessary effect if a bactericidal effect occurs.

In order to analyze the mechanism of antibacterial activity against periodontal disease bacteria, we decided to prepare a synthetic cyclo (Hyp-Leu) (**Figure 4**). The characteristic structure of cyclo (L-Hyp-L-Leu) **1** (**Figure 4**) is its 2,5-diketopiperazine skeleton within the molecule. It has been reported that the 2,5-diketopiperazine skeleton forms by condensation of 2 amino acid derivatives followed by intramolecular cyclization of the dipeptide (Wang et al., 2002). Based on this method, we attempted the synthesis of cyclo (L-Hyp-L-Leu) **1**. We consider that racemization hardly occurs during condensation or intramolecular cyclization, because cyclo (L-Hyp-D-Leu) (or cyclo (D-Hyp-L-Leu)) could not be detected. A cyclic dipeptide is stereospecifically synthesized and effectively forms cyclo (L-Hyp-D-Leu) from L-Hyp and D-Leu derivatives. This time, we could confirm a diastereomer equivalent to H8-2 (**Figure 5**). Since antibacterial properties against periodontal disease bacteria were also confirmed (**Figure 6**), it is possible that this feature could be used to elucidate the mechanism of activity in future.

Furthermore, while considering future clinical applications, safety for mucosal epithelial tissues is an important aspect. In particular, for many dipeptides reports exist, which show the effects of anticancer cells, so there is concern about toxicity to eukaryotic cells. The irritation evaluation model system performed this time is widely used to evaluate short-term toxicity for drugs and cosmetics as an alternative technology to animal experiments. Since the synthetic cyclo (Hyp-Leu) seems to have low toxicity in the stimulation test using this epithelial tissue model, it might be put into practical use from the viewpoint of safety.

In conclusion, the substances isolated from fractions H8-1 and H8-2 of *L. fermentum* ALAL020 culture have antibacterial activity against periodontopathic bacteria *P. gingivalis* and *P. intermedia*, and were both identified as hexahydro-7-hydroxy-3-(2-metylpropyl) pyrrolo [1,2-a] pyrazine-1,4-dion. It is most likely that the slight difference in the fraction peaks of H8-1 and H8-2 was observed because they are steric isomers. Among the previously reported antibacterial substances produced by *L. fermentum*, no report indicates production of this substance, which is a novel finding obtained from this study. We believe that the results of this analysis will be useful for elucidating the production mechanism of this substance and the antibacterial mechanism against periodontal disease bacteria. Further studies are necessary on the mechanism of production and action, as well as the potential for clinical application.

## Data Availability Statement

The original contributions presented in the study are included in the article/**Supplementary Material**. Further inquiries can be directed to the corresponding author.

## Author Contributions

TK designed and coordinated the study, performed the measurement and interpretation of the data. TO contributed to the design and coordination of the study, performed the measurement and interpretation of the data. NM participated in the design and interpretation of the data; deceased Jan. 28, 2020. TT, SI, AT, NI, RS, and YI performed the measurement and interpretation of the data. TO and KM wrote and checked the manuscript. The authors read and approved the final manuscript.

## Funding

The authors acknowledge financial support from the Ministry of Education, Culture, Sports, Science, and Technology (MEXT) of Japan in the form of a Grant-in-Aid for Young Scientists, No. 18K17057.

## Conflict of Interest

Authors RS and YI are employed by the company A. L. A. Corporation, but no-funding was offered to this study from A. L. A. Corporation. In addition, A. L. A. Corporation was not involved in the study design, collection, analysis, interpretation of data, the writing of this article or the decision to submit it for publication.

The remaining authors declare that the research was conducted in the absence of any commercial or financial relationships that could be construed as a potential conflict of interest. 

## Publisher’s Note

All claims expressed in this article are solely those of the authors and do not necessarily represent those of their affiliated organizations, or those of the publisher, the editors and the reviewers. Any product that may be evaluated in this article, or claim that may be made by its manufacturer, is not guaranteed or endorsed by the publisher.
