# Supplementary figures and images for: Limosilactobacillus (Lactobacillus) fermentum ALAL020, a Probiotic Candidate Bacterium, Produces a Cyclic Dipeptide That Suppresses the Periodontal Pathogens Porphyromonas gingivalis and Prevotella intermedia
Source: Front Cell Infect Microbiol. 2022 Mar 7;12:804334. doi: 10.3389/fcimb.2022.804334 (PMC8936145; doi:10.3389/fcimb.2022.804334)

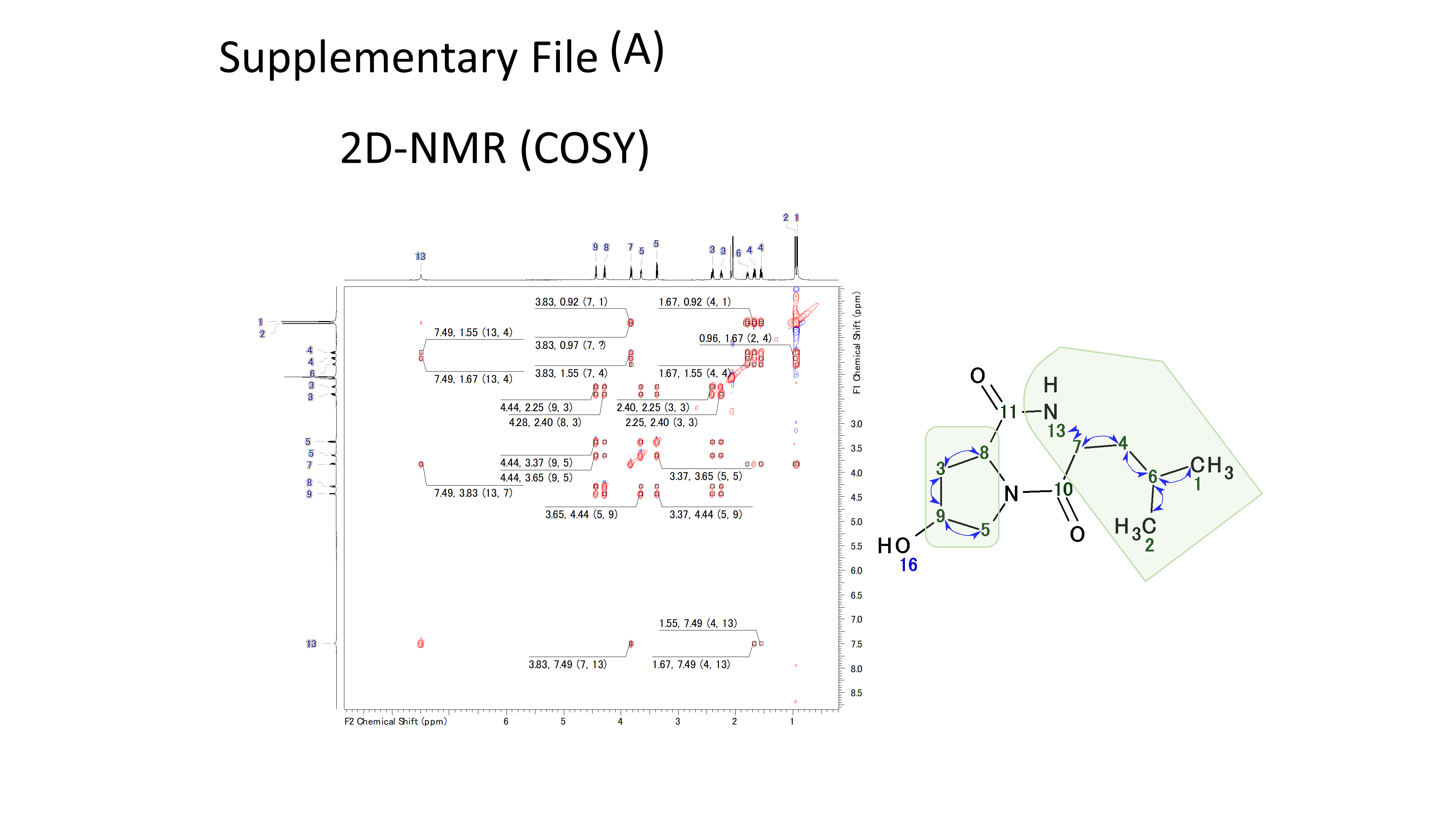

Supplement: Supplementary file 1 [file Image_1.tif]

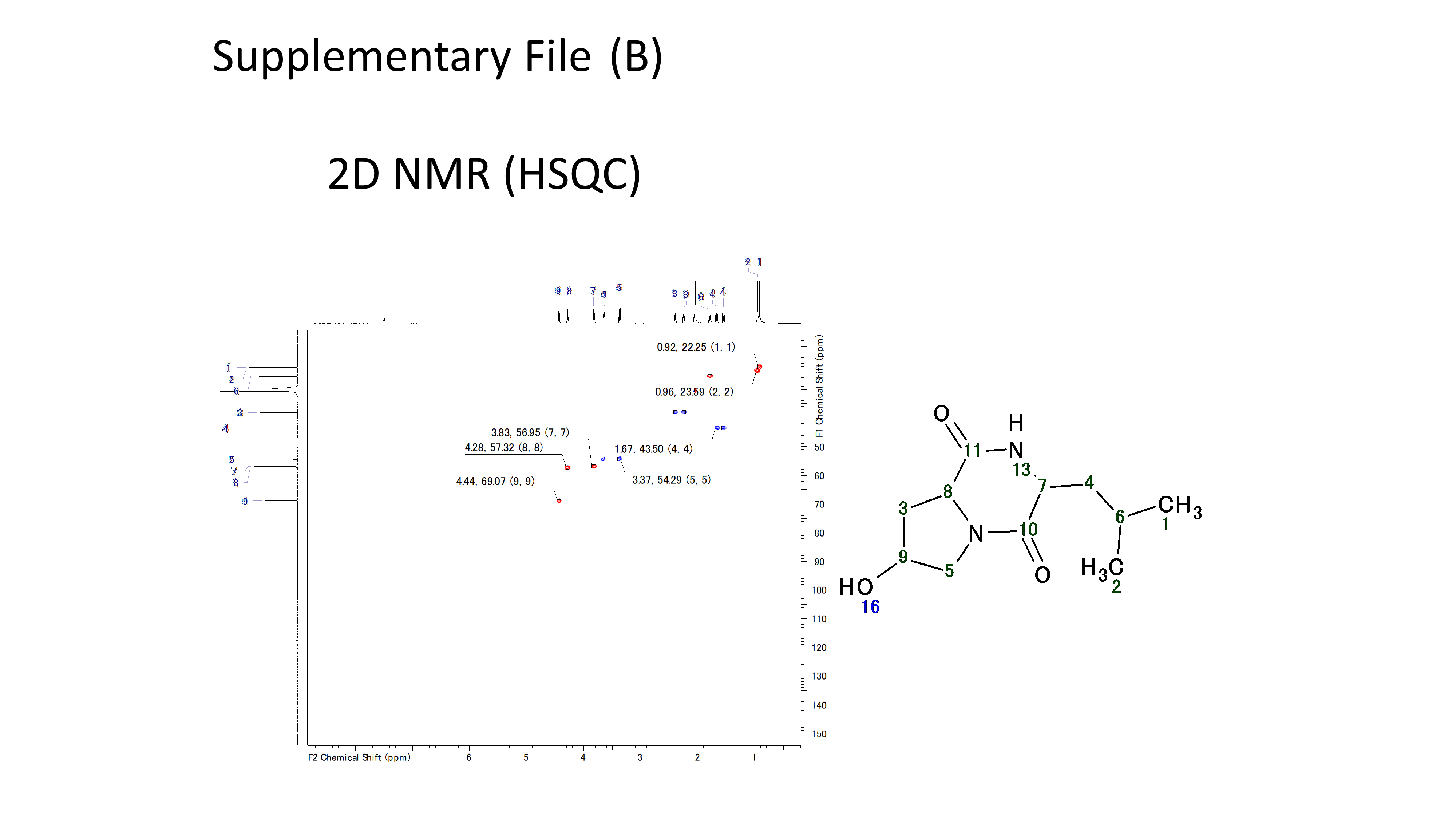

Supplement: Supplementary file 2 [file Image_2.tif]

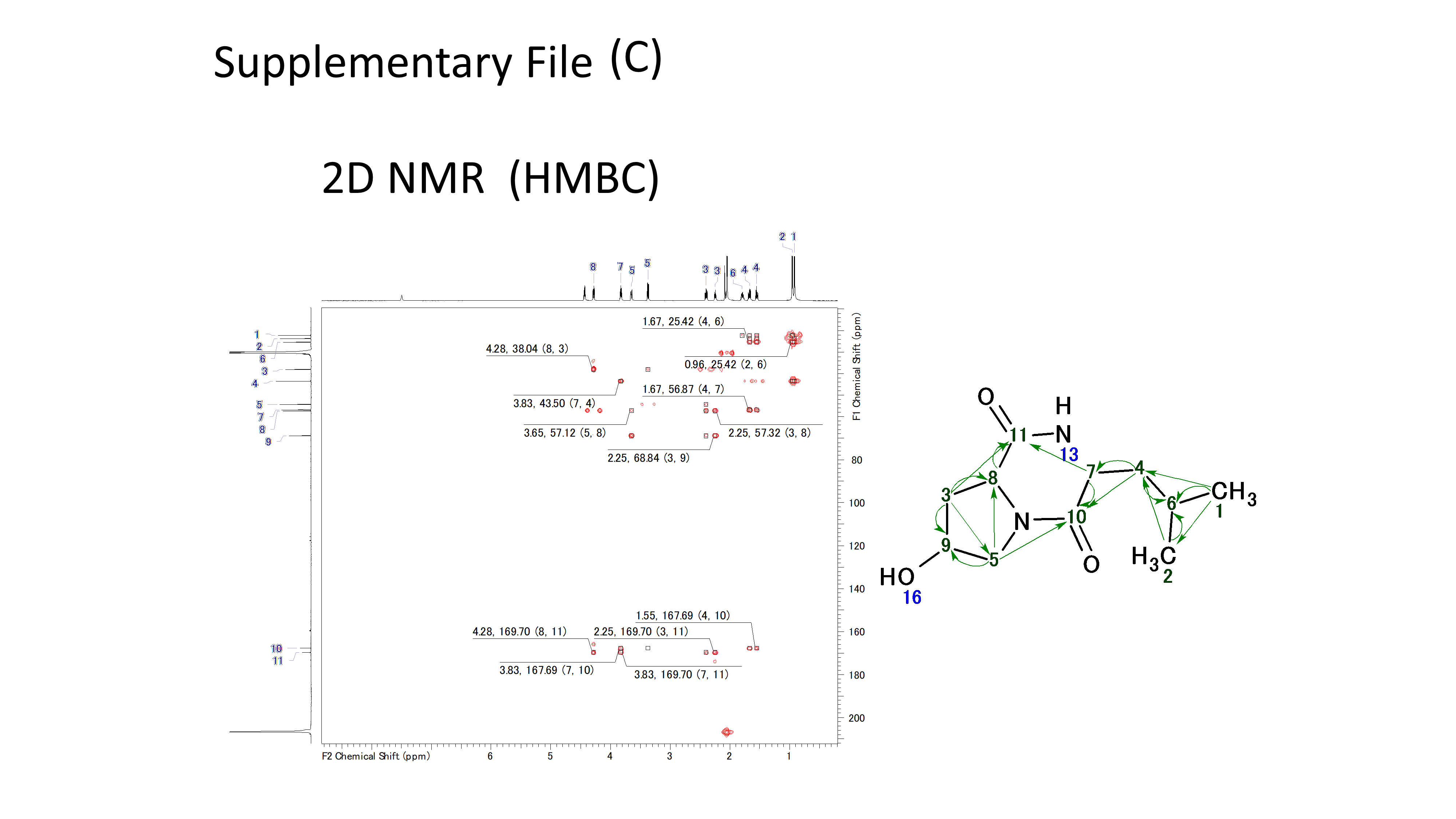

Supplement: Supplementary file 3 [file Image_3.tif]

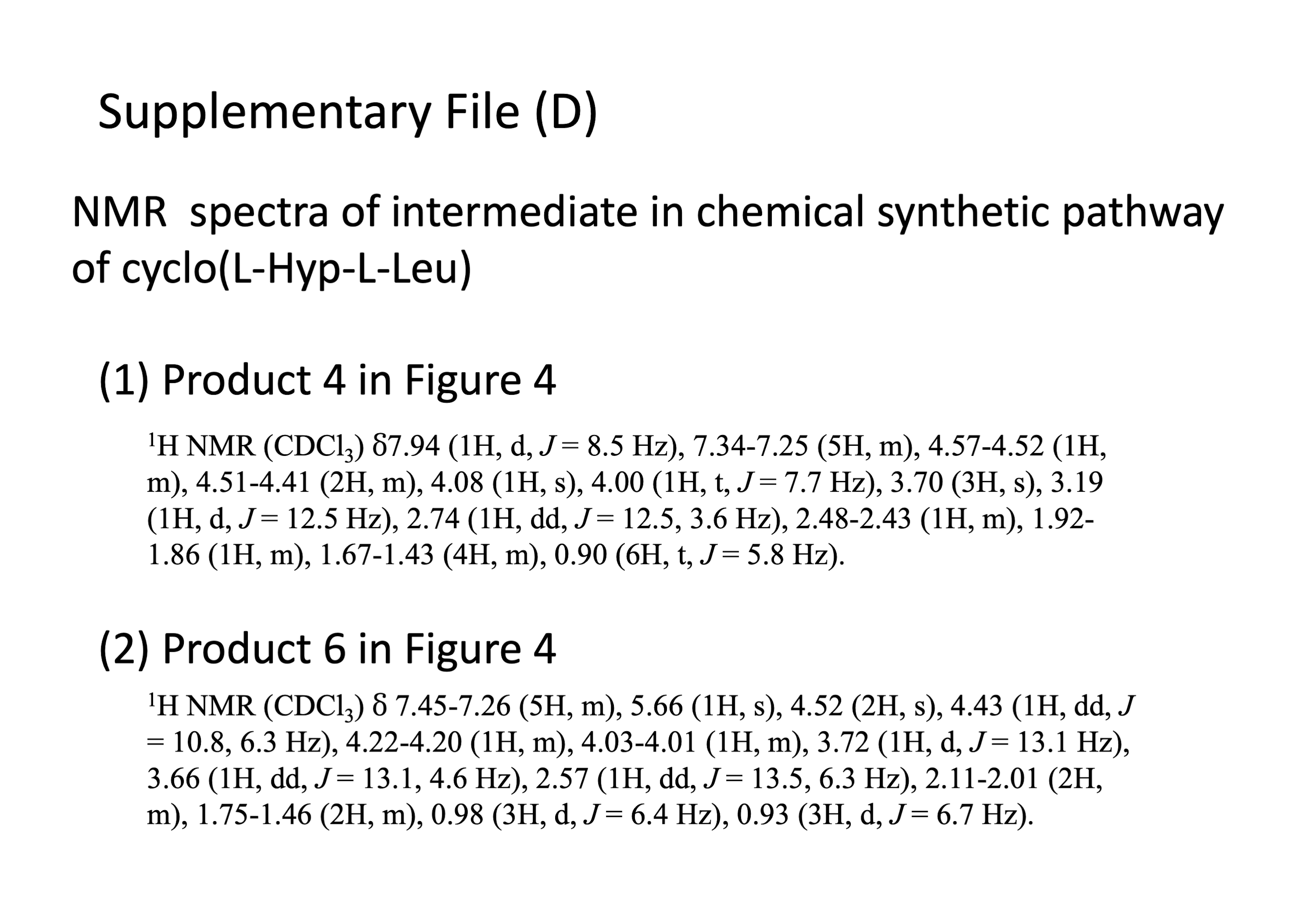

Supplement: Supplementary file 4 [file DataSheet_4.zip › Supplimentary file (D).tiff]

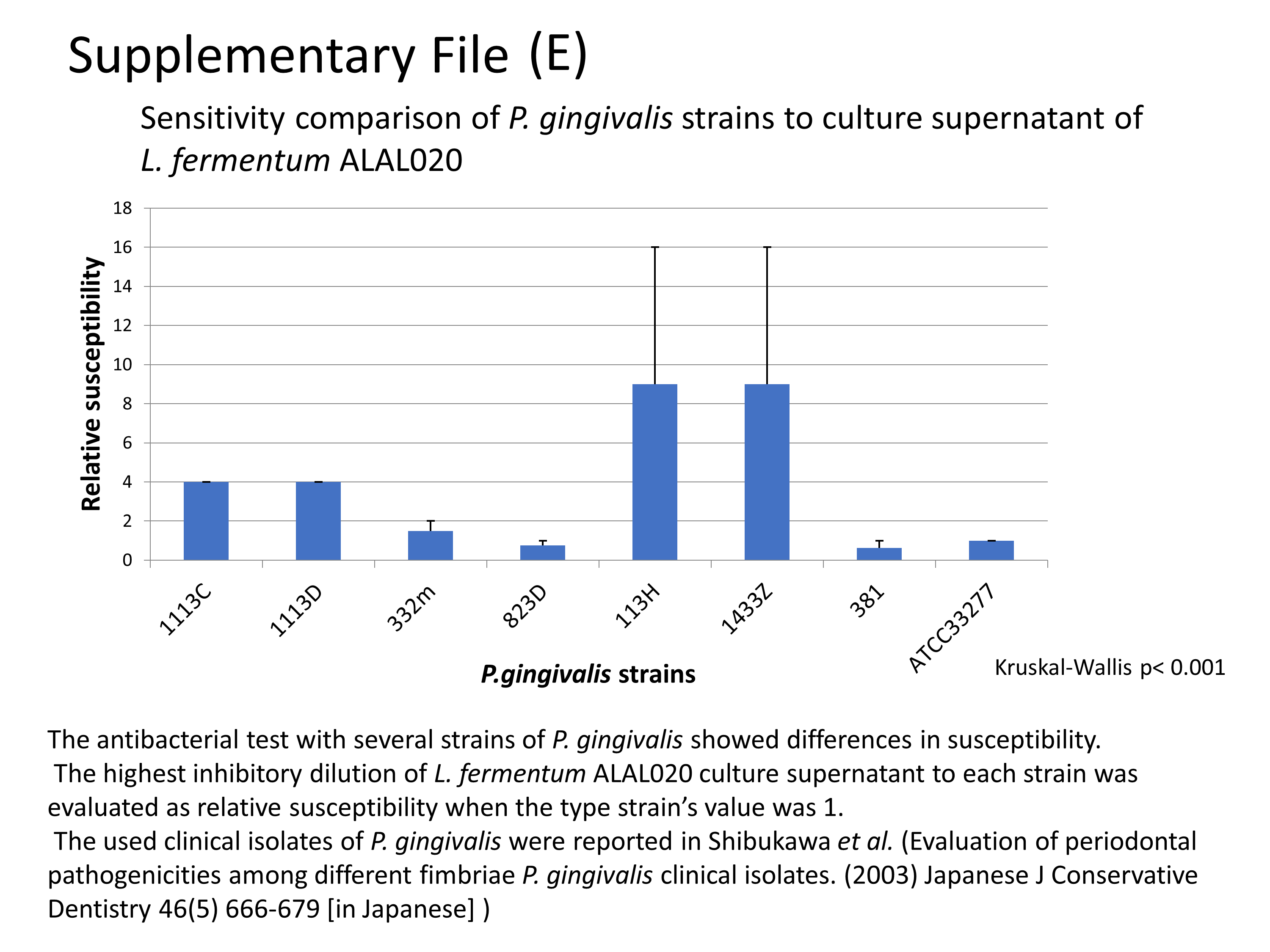

Supplement: Supplementary file 5 [file Image_5.tif]
